# Doubling of coastal flooding frequency within decades due to sea-level rise

**DOI:** 10.1038/s41598-017-01362-7

**Published:** 2017-05-18

**Authors:** Sean Vitousek, Patrick L. Barnard, Charles H. Fletcher, Neil Frazer, Li Erikson, Curt D. Storlazzi

**Affiliations:** 1 University of Illinois at Chicago, Chicago, IL 60607 USA; 2 US Geological Survey, Pacific Coastal & Marine Science Center, Santa Cruz, CA 95060 USA; 3 University of Hawaii at Manoa, Honolulu, HI 96822 USA

## Abstract

Global climate change drives sea-level rise, increasing the frequency of coastal flooding. In most coastal regions, the amount of sea-level rise occurring over years to decades is significantly smaller than normal ocean-level fluctuations caused by tides, waves, and storm surge. However, even gradual sea-level rise can rapidly increase the frequency and severity of coastal flooding. So far, global-scale estimates of increased coastal flooding due to sea-level rise have not considered elevated water levels due to waves, and thus underestimate the potential impact. Here we use extreme value theory to combine sea-level projections with wave, tide, and storm surge models to estimate increases in coastal flooding on a continuous global scale. We find that regions with limited water-level variability, i.e., short-tailed flood-level distributions, located mainly in the Tropics, will experience the largest increases in flooding frequency. The 10 to 20 cm of sea-level rise expected no later than 2050 will more than double the frequency of extreme water-level events in the Tropics, impairing the developing economies of equatorial coastal cities and the habitability of low-lying Pacific island nations.

## Introduction

Global sea level is currently rising at ~3–4 mm/yr^1, 2^ and is expected to accelerate due to ocean warming and land-based ice melt^3, 4^. Sea-level rise (SLR) projections range from 0.3 to 2.0 m by 2100, depending on methodology and emission scenarios^5, 6^, and recent work suggests that accepted methodologies significantly underestimate the contribution of Antarctica^7^.

**Figure 1:**
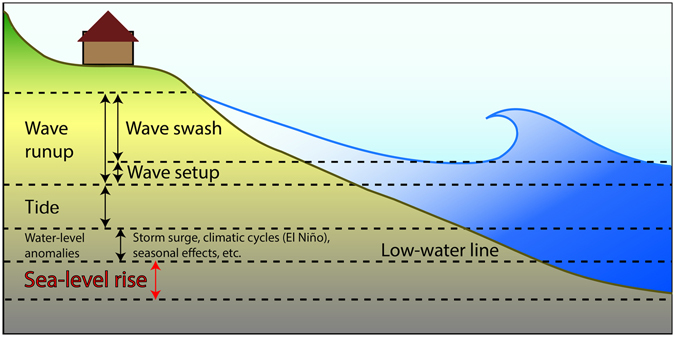
The water-level components that contribute to coastal flooding.

Coastal regions experience elevated water levels on an episodic basis due to wave setup and runup^8^, tides^9^, storm surge driven by wind stress and atmospheric pressure, contributions from seasonal and climatic cycles, e.g., El Niño/Southern Oscillation^10, 11^ and Pacific Decadal Oscillation^12^, and oceanic eddies^13^ (Fig. 1).

Coastal flooding often occurs during extreme water-level events that result from simultaneous, combined contributions, such as large waves, storm surge, high tides, and mean sea-level anomalies^11, 14^.

SLR leads to (1) passive high-tide inundation of low-lying coastal areas^15^, (2) increased frequency, severity, and duration of coastal flooding^16^, (3) increased beach erosion^17^, (4) groundwater inundation^18, 19^, (5) changes to wave dynamics^20^, and (6) displacement of communities^21^. Predicting regions vulnerable to passive inundation is relatively simple with the aid of high-resolution digital elevation models^22^. However, predicting the effect of SLR on episodic flooding events is difficult due to the unpredictable nature of coastal storms, nonlinear interactions of physical processes (e.g., tidal currents and waves), and variations in coastal geomorphology (e.g., sediments, bathymetry, topography, and bed friction). Local-scale assessments of coastal hazard vulnerability typically rely on detailed, computationally-onerous numerical modeling efforts^23^ in order to simulate wave-related nearshore water levels, interactions with local topography, and the resulting flooding. Global-scale coastal hazard vulnerability assessments, on the other hand, rely on extreme value theory applied to water-level observations.

### Extreme-value theory

Extreme-value theory^24, 25^ is a statistical method for quantifying the probability or return period of large events. The generalized extreme value (GEV) distribution, sometimes called the Fisher-Tippet distribution, is a powerful and general statistical model for extremes^26^ (Coles 2001). The GEV distribution models the probabilities of the maxima of a random variable^24, 27, 28^ using three parameters *μ*, *σ*, and *k*, the location (mean), scale (width), and shape (family type), respectively^26^.

**Figure 2:**
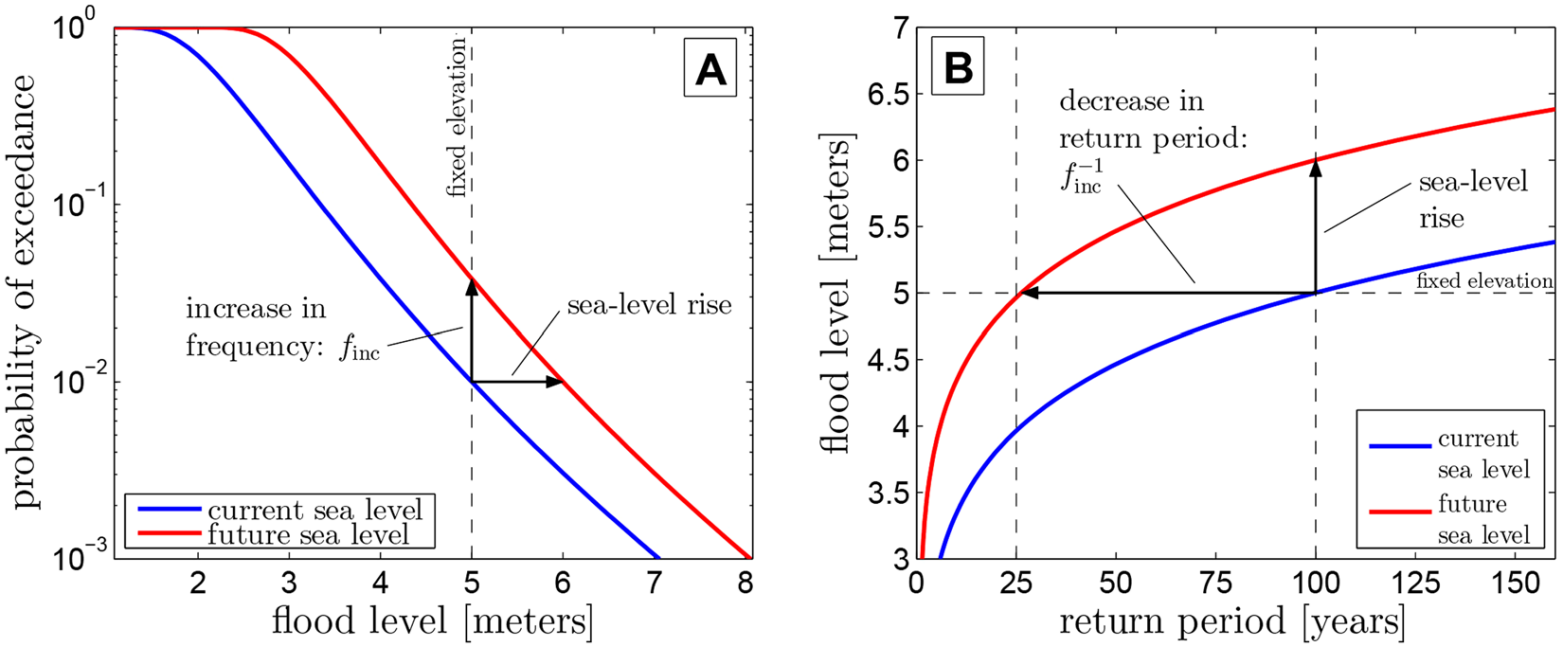
Example: by elevating the exceedance probability distribution, a 1 m increase in SL increases the frequency (**A**) and lowers the return period (**B**) of the 5m-flood level. Note that the steeper the probability distribution in A, the flatter the return time curve in B, i.e., the greater the increase in frequency and the reduction in return time. Thus regions with lower variability in flood level will experience larger increases in flooding frequency under SLR. See Methods and extended data Figs 1 and 2.

Oceanographic and coastal engineering studies often rely on GEV theory to describe the frequency of extreme waves^29^, water-level events^30^, flooding impacts^31^, and to understand the effects of SLR^32^. As sea level increases, the probability increases that a fixed elevation will experience flooding (Fig. 2). Equivalently, the return period or recurrence interval of flooding at a fixed elevation decreases^33, 34^. In the example shown in Fig. 2B, 1 m of SLR causes the 5 m flood level (the former 100-year flood) to recur every 25 years.

SLR can affect flood magnitude and frequency directly (Fig. 2) or indirectly via hydrodynamic feedbacks: SLR alters water depths, changing the generation, propagation, and interaction of waves, tides, and storm surges. Thus, SLR and long-term changes in wave climate, e.g., changes in magnitude, frequency, and tracks of storms^35–37^ and storm surge, can alter the parameters of extreme water-level distributions and the evolution of coastal hazards over time. In the proposed work, we assume parameter stationarity based on projections of minor changes (5–10%^35–37^) in mean annual wave conditions and storm surge over large regions of the ocean. In specific locations, such as the Pacific Northwest, trends in extreme wave climate may be significant^38^ and lead to a greater flooding hazard than SLR over at least the next several decades^39^, calling for nonstationary methods^40^ in future research.

Investigations of increased flooding frequency due to SLR are often site-specific and rely only on water-level data from tide stations. For example, Hunter (2012) [ref. 41] and the Intergovernmental Panel on Climate Change (IPCC) 2013 report^3^ estimate the factor of increase in the frequency of flooding events due to 0.5 m of SLR at locations of 198 tide stations around the globe [Hunter^41^ Fig. 4 and IPCC^3^ Fig. 13.25]. Hunter^41^ and IPCC^3^ found that regions with low variability of extreme water levels will experience large increases in flooding frequency. This finding, introduced qualitatively by Hoozemans *et al*. [ref. 33], is critical to predict the global regions most vulnerable to SLR. However, global-scale coastal hazard assessments using this methodology encounter three challenges: (1) Water-level observation stations are sparsely located around the globe, especially in the Indian Ocean and South Atlantic; (2) wave-driven water-level contributions, i.e., setup and swash, are not included; and (3) the global variability of the GEV shape parameter has not been considered, although it can be as influential as the scale parameter in determining vulnerability. Here we meet the three challenges by using extreme-value theory to combine sea level, wave, tide, and storm-surge models to predict increases in extreme water-level frequency on a global scale.

### Application

Flooding results from the complex interaction of extreme water levels, topography, and the built environment. Here we use the frequency of extreme water levels as a proxy for regional-scale increases in flooding frequency, while recognizing that the relationship between water level and flooding is location dependent because of coastal topography, coastal defense structures, and drainage systems.

We apply sea-level projections and global wave, tide, and storm surge models to predict the future return periods (associated with the former 50-yr extreme water level) due to SLR. As in Hunter^41^ and IPCC^3^, we begin by investigating increases in flooding frequency due to a globally-uniform amount of SLR, acknowledging that spatial variability in the regional rate of SLR (e.g., driven by ocean circulation patterns, glacial fingerprinting) and the local relative rate of SLR (e.g., due to tectonic activity, glacial isostasy, land subsidence) will affect flooding predictions for specific locations^42^. Later we take the inverse approach, estimating the amount of SLR that doubles the frequency of extreme water-level events.

**Figure 3:**
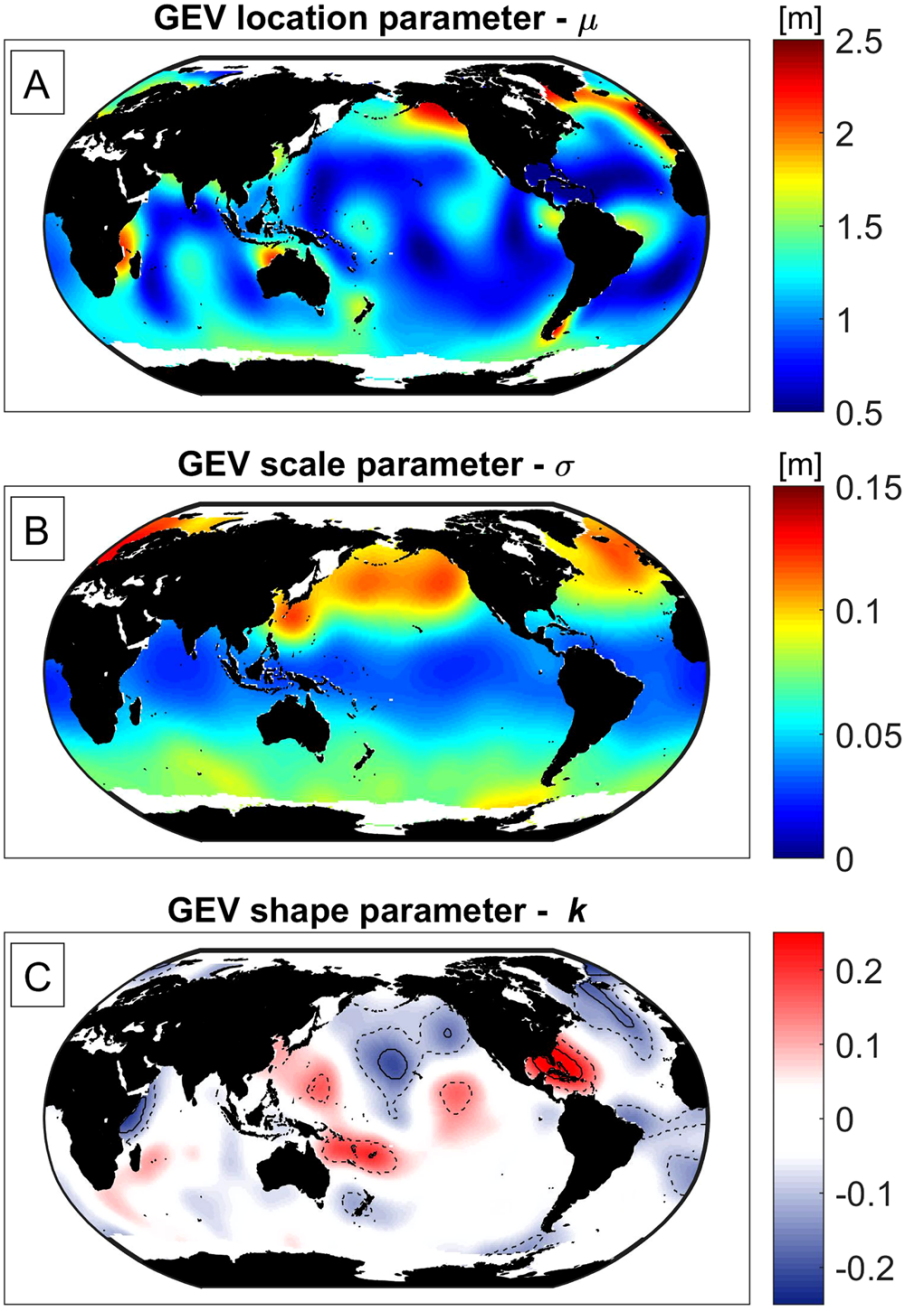
Global estimates of the location (*μ*), scale (*σ*), and shape (*k*) parameters of the GEV distribution of extreme water-level (the sum of wave setup, tide, and storm surge) shown in panels A, B, and C, respectively. The dashed and solid lines in panel C represent contours of *k* that are significantly different from zero at the 75% and 95% confidence levels, respectively. The maps in this figure were made using Matlab 2016a (https://www.mathworks.com/products/matlab/).

**Figure 4:**
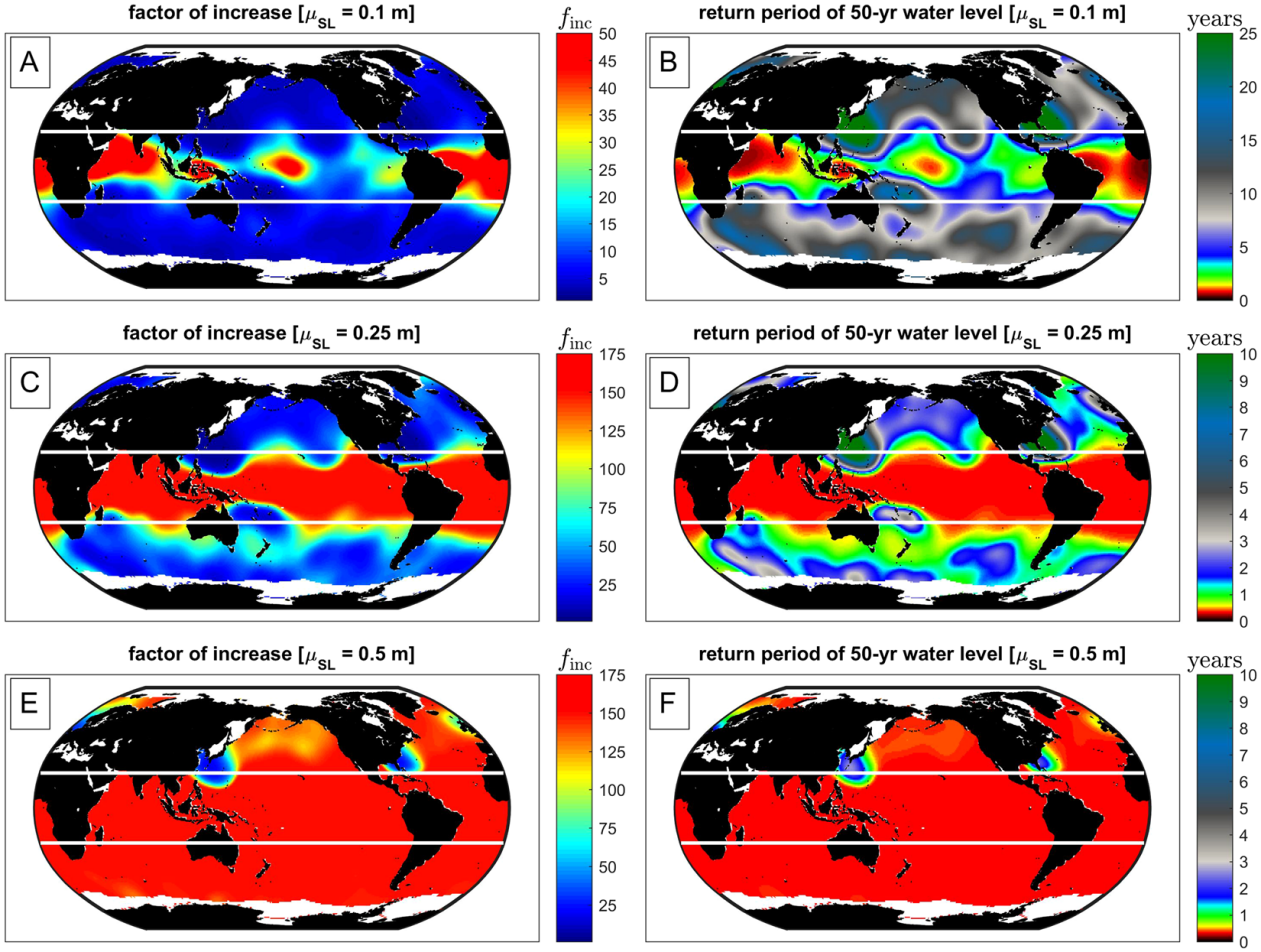
Global estimates of the expected factor of increase in exceedance probability, *f*_*inc*_, and the future return period, *T*_*R*_, of the 50-yr water level, for SLR projections: *μ*_*SL*_ = +0.1, +0.25, +0.5 m. We note that the estimated increase in flooding potential is purely due to SLR and not due to changes in climate or storminess. White lines indicate the Tropic of Cancer and Tropic of Capricorn. The maps in this figure were made using Matlab 2016a (https://www.mathworks.com/products/matlab/).

**Figure 5:**
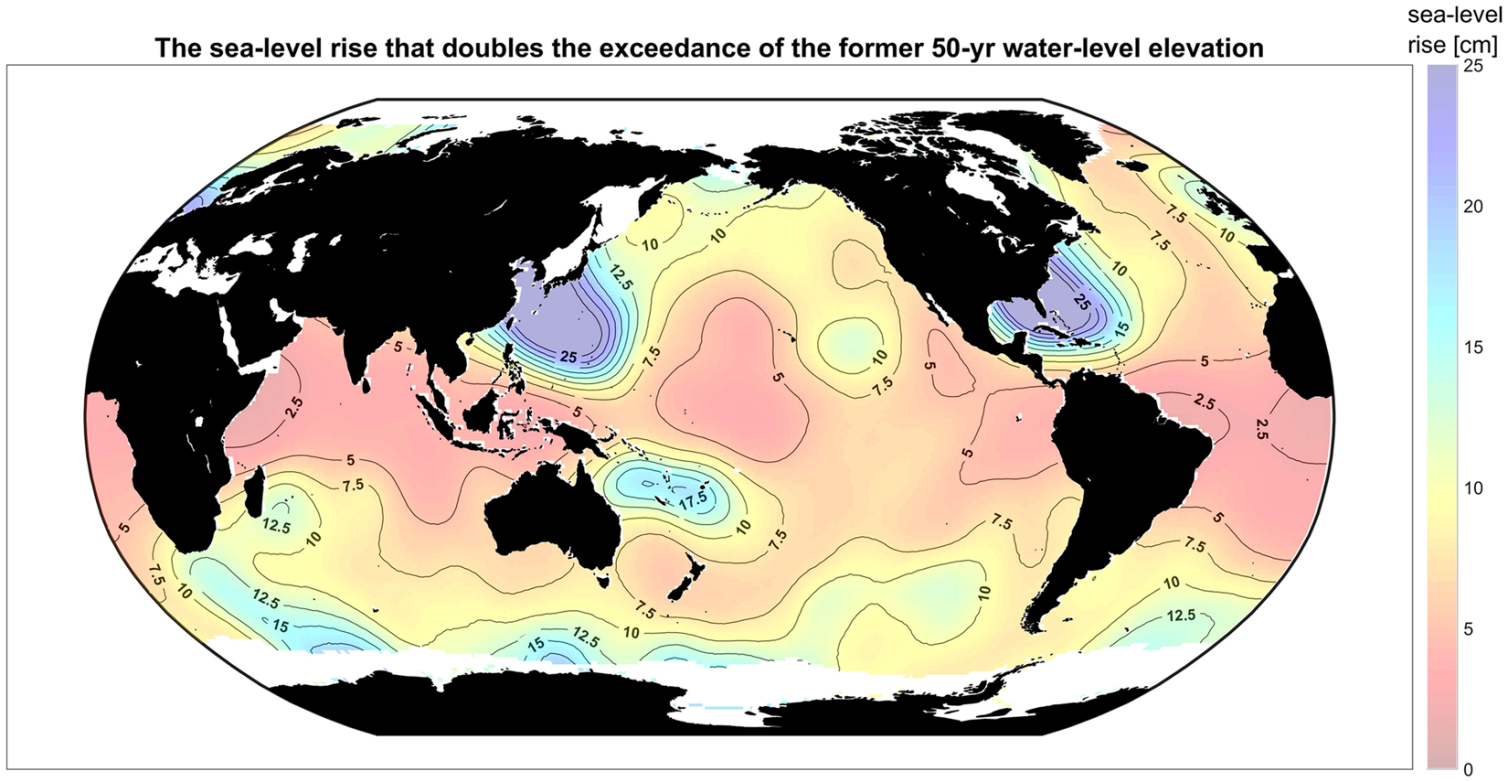
The upper bound of SLR that doubles the exceedance probability of the former 50-year water level. This SLR is the upper limit of a 95% confidence interval based on a Monte Carlo simulation of the GEV parameter estimates and their associated confidence bands (see Methods). Red areas represent regions particularly vulnerable to small amounts of SLR. The maps in this figure were made using Matlab 2016a (https://www.mathworks.com/products/matlab/).

Using maximum likelihood estimates, we fit GEV probability distributions to the top three annual maximum water-level events from 1993–2013 obtained via synthesis of the Global Ocean Wave (GOW) reanalysis^43^, Mog2D storm-surge model^44^, and TPXO tide model^45^ as discussed in Methods. Figure 3 shows the global variability of the mean (*μ*), scale (*σ*), and shape (*k*) parameters for extreme total water level in panels A, B, and C, respectively. The GEV parameters provide necessary inputs to the factors of increase, *f*_*inc*_, and the future return period of the former 50-yr water level based on Eq. (3) (see Methods). Figure 4 shows the factor of increase for the SLR projections *μ*_*SL*_ = +0.1, +0.25, +0.5 m on a global scale. Finally, the GEV parameters allow for global estimation of the amount of SLR that doubles the exceedance probability of the 50-yr water-level elevation [see Fig. 5 and Methods Eq. (4)]. Analyzing the amount of SLR leading to a doubling in flooding (Fig. 5) is equivalent to the factor-of-increase results shown in Fig. 4, but it provides a more intuitive picture of the effects of small amounts of SLR. Table 1 summarizes the global, tropical, and extra-tropical mean values of the quantities presented in Figs 3 and 5. Although the plotted distributions apply only to coasts, they are calculated ocean-wide in order to reveal the continuous global pattern of vulnerability of both continental coastal settings and non-contiguous island nations throughout the world’s oceans.

**Table 1:** Mean values of GEV parameters (Fig. 3), factors of increase (Fig. 4), and doubling SLR (Fig. 5) in the tropics, extratropics, and worldwide.

	Tropics	Extratropics	Global mean
Location parameter, *μ*	0.97 m	1.16 m	1.09 m
Scale parameter, *σ*	0.04 m	0.08 m	0.06 m
Shape parameter, *k*	0.00	−0.04	−0.02
*f*_inc_ (SLR = 0.1 m)	24.6	5.5	12.8
*f*_inc_ (SLR = 0.25 m)	127.6	45.3	76.9
*f*_inc_ (SLR = 0.5 m)	147.7	141.3	143.7
*T*_*R*_ of former 50-yr water-level elevation (SLR = 0.1 m)	4.9 yrs	10.9 yrs	8.6 yrs
*T*_*R*_ of former 50-yr water-level elevation (SLR = 0.5 m)	0.74 yrs	1.8 yrs	1.4 yrs
*T*_*R*_ of former 50-yr water-level elevation (SLR = 1.0 m)	0.35 yrs	0.38 yrs	0.37 yrs
Doubling sea-level, *μ*_2x_, for the 50-yr water-level elevation (expected value)	2.9 cm	4.6 cm	3.9 cm
Doubling sea-level, *μ*_2x_, for the 50-yr water-level elevation (upper bound of 95% confidence interval)	6.5 cm	9.9 cm	8.6 cm

## Discussion

We first consider the GEV parameters for extreme water levels (Fig. 3), then the frequency increases (Fig. 4), followed by the SLR threshold that doubles exceedance of the 50-yr water level (Fig. 5).

The spatial variability in the GEV location parameter (*μ*) is shown in Fig. 3A. Globally, 99% of the values of *μ* fall between 0.50 and 2.13 m. The location parameter strongly resembles the M_2_ tidal amplitude^45^ yet is also influenced by global wave climate. The parameter is largest in the North Pacific and North Atlantic due to large tides and the occurrence of extratropical storms that track mainly west to east, producing large, latitudinally-isolated waves. The scale parameter (*σ*) ranges from 0.024 to 0.118 m (Fig. 3B) and is correlated to the location parameter with *r* = 0.47. In other words, the regions that experience the largest water levels also experience the largest variance in those levels. The spatial variability of the shape parameter (*k*) is uncorrelated with that of the other GEV parameters.

The shape parameter ranges from −0.18 to 0.20 (Fig. 3C) with a global mean of −0.024. Notably, the geographic regions in Fig. 3C with large (positive) values of the shape parameter are regions with high densities of tropical storm tracks, i.e., the Tropics and lower mid-latitudes of the western Pacific and Atlantic Oceans. The range and geographic variability of the shape parameter in Fig. 3C is remarkably similar to previously reported results for the shape parameter of extreme wave heights^46^, underscoring the importance of wave-driven water-level components (See Extended Data Figs 3 and 8 for details) and the role of tropical cyclones on the magnitude and spatial distribution of the shape parameter.

In theory, negative values of the shape parameter, i.e., bounded water-level distributions, are expected based on the notion that upper bounds on tide, storm surge, and maximum wave heights exist due to limiting processes (e.g., wave breaking and physical limits in wind speed, fetch, and duration prevent unbounded wave heights). On the other hand, positive values of the shape parameter, i.e., unbounded water-level distributions, indicate the probability of exceedingly large yet inconsistent water-level events relative to an annual event. In practice, both positive and negative values of the shape parameter are possible because of the limited amount of data available for parameter estimation and the possibility of outliers. Thus, it is difficult to assess, a priori, whether the large values of the shape parameter result from a proper characterization of the variability of tropical cyclones or from the presence of outliers among a temporally-limited data set. We expect that more than 21 years of data (used here) would likely improve the characterization of extreme events due to tropical cyclones and the estimation of the shape parameter.

The dashed and solid lines in panel C (Fig. 3) represent contours of *k* that are significantly different from zero at the 75% and 95% confidence levels, respectively. The near-zero mean and the limited extent of the statistically significant non-zero values of the shape parameter in Fig. 3C suggests that the Gumbel distribution [the GEV family when *k* = 0, as in Hunter^41^ and IPCC^3^] might suffice for global-scale assessments of SLR impacts. However, for smaller-scale regions of interest, particularly the Caribbean Sea, the Central North Pacific, and North Atlantic, the variability of the shape parameter should be accounted for when predicting the effects of SLR.

Next, we discuss how the global GEV parameters characterize the increased frequency of flooding due to SLR (Figs 4 and 5). Although the behavior of the scale parameter is well known [as introduced by Hoozemans *et al*.^33^, and further explored in Hunter^41^ and IPCC^3^], these figures provide the first continuous, global demonstration of that behavior, as well as the first incorporation of wave-driven water levels.

The factor of increase in frequency of the 50-yr extreme water-level event, *f*_*inc*_, and the future return period of the former 50-yr extreme water level due to SLR, $50\,{f}_{{inc}}^{-1}$, are shown in Fig. 4. For fixed SLR, decreasing values of the scale and shape parameters increase *f*_*inc*_ and thus reduce the return period of the present 50-yr water level. The increase in *f*_*inc*_ is larger in the Tropics (white lines on Fig. 4) compared to the Extratropics. The results presented in Fig. 4 and Table 1 indicate that the average factor of increase in flooding, *f*_*inc*_, in the Tropics with only 10 cm of SLR is approximately 25 times present levels, and the former 50-yr event occurs every 4.9 years. Outside the Tropics, the average factor of increase is 5.5, and the former 50-yr event occurs every 10.9 years. Note that the results given in Table 1 do not exactly follow the reciprocal relationship between the increase in frequency (*f*_*inc*_) and the reduction in return period ($50\,{f}_{{\rm{inc}}}^{-1}$) because of the spatial averaging operation. Finally, we note that the estimated increase in flooding potential is purely due to SLR and not due to possible future changes in wave climate or storm patterns.

The upper bound of the doubling SLR, *μ*_2x_, (Fig. 5) is estimated as the upper limit of the 95% confidence intervals of the GEV parameter estimates using Eq. (4) in Methods. As shown in Fig. 5, only 5–10 cm of SLR, expected under most projections to occur between 2030 and 2050 ^5^, doubles the flooding frequency in many regions, particularly in the Tropics, and would occur even more rapidly in areas where regional SLR exceeds the eustatic rate^12^. Less than 5 cm of SLR doubles the frequency of the 50-yr water level in the tropical Atlantic and northwestern Indian Ocean. The maps of increased flooding potential (Figs 4 and 5) suggest a dire future for the top 20 cities (by GDP) vulnerable to coastal flooding due to SLR^47^, and for many wave-exposed cities such as Mumbai, Kochi, Grande Vitoria, and Abidjan which may be significantly affected by only 5 cm of SLR. Less than 10 cm of SLR doubles the flooding potential over much of the Indian Ocean, the south Atlantic, and the tropical Pacific. Only 10 cm of SLR doubles the flooding potential in high-latitude regions with small shape parameters, notably the North American west coast (including the major population centers Vancouver, Seattle, San Francisco, and Los Angeles), and the European Atlantic coast. The only regions where 15 cm of SLR does not double the flooding potential are regions with large shape parameters (likely influenced by tropical storm tracks): the mid-latitudes of the northwestern Pacific below Japan, the mid-latitudes of the northwestern Atlantic (the U.S. east coast, Gulf of Mexico, and Caribbean Sea), and the southwest tropical Pacific encompassing Fiji and New Caledonia (discussed below).

The Tropics experience limited water-level variance due to consistently smaller wave heights (due to latitudinal gradients in storm activity) and smaller tide ranges (due to the presence of tidal amphidromes) throughout the region. Consequently, SLR represents a larger percentage of the water-level variance as explained in Fig. 2 and Methods. The mid-latitudes of the northwestern Pacific and the northwestern Atlantic experience smaller increases in extreme water-level frequency due to large values of the scale and shape parameter, respectively. Notably, the mid-latitudes of the northwestern Pacific below Japan experience large values of the scale parameter without correspondingly large values of the location parameter as in most of the north Pacific and north Atlantic, possibly due to the consistency of tropical storms in the region. The mid-latitudes of the northwestern Atlantic (e.g., the U.S. east coast, Gulf of Mexico, and Caribbean Sea), on the other hand, have elevated values of the shape parameter due to the intermittent occurrence of tropical cyclones, which correspond to elevated probabilities of large extremes rather than bounded extremes. This suggests that although the continued and accelerating impacts of SLR-driven nuisance flooding is a major concern in many of these areas^16^, the rare occurrence of extreme events (e.g., hurricanes) – and not SLR – will remain the dominant hazard on wave-exposed coastlines in the lower mid-latitudes of the western Pacific and Atlantic for several decades.

## Conclusions

Regions with limited variability in extreme water levels, such as the Tropics, will experience greater increases in flooding frequency due to SLR than regions with significant water-level variability, e.g., the Extratropics. Small amounts of SLR, e.g., 5–10 cm, may more than double the frequency of extreme water-level events in the Tropics as early as 2030. This is an especially critical finding as numerous low-lying island nations in the Tropics are particularly vulnerable to flooding from storms today, and a significant increase in flooding frequency with climate change will further challenge the very existence and sustainability of these coastal communities across the globe^48^.

## Methods

### Generalized Extreme Value (GEV) distribution

The cumulative distribution function (CDF) of the Generalized Extreme Value (GEV) distribution is given by

1 $$F(x;\mu ,\sigma ,k)=\{\begin{array}{c}{e}^{-{(1+k(\frac{x-\mu }{\sigma }))}^{-1/k}}\,{\rm{for}}\,k\ne 0\\ {e}^{-{e}^{-(\frac{x-\mu }{\sigma })}}\qquad \quad {\rm{for}}\,k=0\end{array}$$

where *F* is the probability that water level *x* will not be exceeded in any one-year period, and *μ*, *σ*, and *k* are the location, scale, and shape parameters, respectively^26^. The GEV distribution includes as special cases three families of extreme value distributions: Gumbel (type I), Fréchet (type II) and Weibull (type III), corresponding to values of the shape parameter *k* = 0, k > 0, and *k* < 0, respectively. Depending on the value of the shape parameter, *k*, the support of *F*(*x*) is either the entire real axis when *k* = 0 or $\{x:1+k(x-\mu )/\sigma  > 0\}$ when *k* ≠ 0^26^. From Eq. (1), the exceedance probability distribution, i.e., the probability that water level *x* is exceeded in any one-year interval, is *E* = 1−*F*. Thus *E*(*x*) is the expected frequency (with units of years^−1^) of events exceeding *x*. The return period, *T*_*R*_, or expected time-interval between events of level *x* or greater is therefore

2 $${T}_{R}=1/E(x),$$

with units of years. For example, a 100-year event has an exceedance probability of 0.01, that is, a 1% chance of occurring in any year. Although return period carries exactly the same information as exceedance probability, it is often more intuitive.

The factor of increase in exceedance probability for SLR *μ*_SL_ > 0 relative to a baseline (*μ*_SL_ > 0) is given by

3 $${f}_{{\rm{inc}}}(x;\mu ,{\mu }_{{\rm{SL}}},\sigma ,k)=\frac{E(x;\mu +{\mu }_{{\rm{SL}}},\sigma ,k)}{E(x;\mu ,\sigma ,k)},$$

and the factor of decrease in return period is ${f}_{{\rm{inc}}}^{-1}$. For example, for the 50-yr event, ${T}_{R}(x;\mu ,\sigma ,k)=50$ years, hence the future return period of the former 50-yr water-level elevation is $50{f}_{{\rm{inc}}}^{-1}$ as shown in Fig. 4B,D and F.

Finally, we reframe the extreme value analysis to determine the amount of SLR leading to a doubling in exceedance of a particular water-level elevation. Note that in Fig. 2, the SLR leading to a 4x increase in probability of the former 100-yr event (e.g., the 25-yr event with +1.0 m of SLR), is simply the difference between the 100-yr water level, $x({T}_{R}=100;\mu ,\sigma ,k)$, and the 25-yr water level, $x({T}_{R}=25;\mu ,\sigma ,k)$, of the unaltered distribution. Thus, the doubling SLR is given by

4 $${\mu }_{2{\rm{x}}}({T}_{R})=x({T}_{R};\mu ,\sigma ,k)-x(\tfrac{1}{2}{T}_{R};\mu ,\sigma ,k)$$

For the example shown in Fig. 5, we use *T*_*R*_ = 50 years. Note that the magnitude of *μ*_2x_ in Eq. (4) and Fig. 5 is controlled by the gradient of the return time function *x*(*T*_*R*_), as explained in Fig. 2B, and that that gradient is controlled by the scale and shape parameters. For low-gradient return time functions, the difference in *x* for the 50 and 25-yr return times is small, and in Fig. 5 the gradient is low for all levels exceeding that of the 10-yr event.

### Application

Well-validated global tide^45^, wave^43^, and storm surge^44^ reanalysis models, each with different spatial and temporal resolutions, are interpolated onto a consistent 1° × 1° grid with hourly time resolution and their water-level components are summed to provide a time series of total water level (TWL). In the proposed approach, we ignore mean sea-level anomalies (MSLA) due to seasonal effects and climate cycles (e.g., El Niño), which, for example, can raise sea level by more than 20 cm along the US west coast^11^, yet are typically less than 20 cm over much of the globe. Large-scale storm surge due to extratropical storms is included in the analysis, but the coarse resolution of the water-level model^44^ precludes simulation of large, spatially isolated hurricane storm surge. On the other hand, the wave fields emanating from hurricanes and tropical cyclones have considerably larger spatial extents and, therefore, are well resolved by the wave model^43^ apart from the near-field generation regions. We limit the time scales considered in our investigation due to the availability of only 21 years of coincident data for waves, tides, and storm surge: extrapolation of 21 years of data to predict 100-year and longer return period events is often problematic.

Hourly time series of tidal water level are computed from 13 harmonic constituents provided by the TPXO tidal inversion model^45^ with native resolution of 0.25° × 0.25° linearly interpolated onto a global grid of 1° × 1°. Time series of wave setup are estimated using the empirical relationship for the 2% exceedance runup on dissipative beaches^8^

5 $${R}_{{\rm{setup}}}=0.016\sqrt{{H}_{0}{L}_{0}},$$

where *H*_0_ and *L*_0_ are the deep-water wave height and wavelength, respectively. We exclude wave swash, the time-varying components of wave runup at incident and infragravity frequencies, because of the large uncertainties associated with the estimation of swash magnitude. For example, wave swash is sensitive to local geological characteristics, notably the beach slope. Wave swash is a time-dependent process, which may or may not affect persistent flood levels. In certain locations, wave swash can significantly contribute to persistent coastal flooding via overtopping of seawalls. Therefore, we include the contribution of wave swash to TWL in Extended Data Figures 5, 6 and 7, which depict the same analyses shown in Figs 3, 4 and 5 (which do not include wave swash). In Extended Data Figures 5, 6 and 7, the magnitude of the 2% exceedance wave swash is estimated using the empirical relationship for dissipative beaches^8^ given by

6 $${R}_{{\rm{swash}}}=0.027\sqrt{{H}_{0}{L}_{0}}$$

which is approximately 1.69 times larger than the wave setup component, Eq. (5). We note that dissipative beach conditions are assumed for the wave runup components in Eqs (5) and (6) in order to avoid the dependence on beach slope.

Time series of *H*_0_ and wave period (*T*) are obtained via the hourly 1° × 1.5° Global Ocean Wave (GOW) reanalysis^43^, and linearly interpolated onto a 1° × 1° grid. The time series of wavelength *L*_0_ = *gT* ^2^/(2*π*) is calculated using linear wave theory from the time series of wave period. Time series of storm surge are obtained from the Mog2D barotropic model^44^ with native resolution of 0.25° × 0.25° at 6-hour intervals, interpolated to an hourly dataset with 1° × 1° resolution. The resulting hourly time series of wave setup, storm surge, and tidal water level for each 1° × 1° grid cell are summed to produce an hourly time series of total water level from 1993–2013. Nonlinear interactions between tide, surge, and wave-driven water levels are not accounted for using this approach. However, processes such as tide-surge interactions may be important in coastal regions around the globe, particularly those adjacent to continental shelves or shallow bathymetry^49^. In general, tides provide the dominant contribution (51% on average) to the total water level (see Extended Data Fig. 3). However, when wave swash is included, wave runup (i.e., wave setup + wave swash) provides the dominant contribution (66% on average) to the total water level (see Extended Data Fig. 8).

Next, GEV distributions are fitted to the top three (*r* = 3) annual maxima (*n* = 63) of the 21-year time series of total water level at each grid point to obtain spatially-varying estimates of the parameters *μ*, *σ*, and *k*. This approach, called the *r*-largest order statistic model, is consistent with the GEV distribution for block maxima^26^. To avoid the case where the *r*-highest values were taken from successive hours, a minimum peak separation criterion of 12 hours was applied. This criterion ensures that the block maxima are independent as required by the *r*-largest order statistic model^26^. The spatial variability of the GEV parameters is smoothed using a penalized least-squares method^50^. Data on the GEV parameter estimates and confidence intervals are available online (see “GEV_data.xlsx”). The GEV parameters *μ*, *σ*, and *k* control the factor of increase *f*_*inc*_ and the future 50-yr return period $50\,{f}_{{\rm{inc}}}^{-1}$ based on Eq. (3), for different values of SLR and event level *x*. Here we set *x* to be the 50-yr water-level event; however the behavior is consistent across a range of extreme values for *x*, particularly those exceeding the 10-yr water level as noted above. Finally, we calculate the sea-level rise, *μ*_2x_, that doubles the exceedance of the former 50-yr water-level elevation based on Eq. (4). To account for the uncertainty in the GEV parameter estimates, a Monte Carlo simulation with 100,000 realizations is applied for each grid point. Each realization generates random values of *μ*, *σ*, and *k* based on the 95% confidence intervals arising from the maximum likelihood estimates and applies Eq. (4) to calculate *μ*_2x_. Next, the upper bound of the doubling sea level (Fig. 5) is calculated as the 95% cumulative probability (%5 exceedance probability) for the empirical distribution of *μ*_2x_. Figure 5 shows the upper end of the 95% confidence level for the SLR that will double (or more than double) the frequency of the 50-yr water-level event.

## Electronic supplementary material

Supplementary info

Dataset 1

Dataset 2
