# Supplementary material for: Doubling of coastal flooding frequency within decades due to sea-level rise
Source: Sci Rep. 2017 May 18;7:1399. doi: 10.1038/s41598-017-01362-7 (PMC5437046; doi:10.1038/s41598-017-01362-7)
Supplement: Supplementary file 1 — Supplementary info [file 41598_2017_1362_MOESM1_ESM.doc]

**Doubling of coastal flooding frequency within decades due to sea-level rise**

By Sean Vitousek^1^, Patrick Barnard^2^, Charles Fletcher^3^, Neil Frazer^3^, Li Erikson^2^, Curt Storlazzi^2^

1. University of Illinois at Chicago, Chicago, IL

2. U.S. Geological Survey, Pacific Coastal & Marine Science Center, Santa Cruz, CA

3. University of Hawaii at Manoa, Honolulu, HI

**Extended Data Figures**


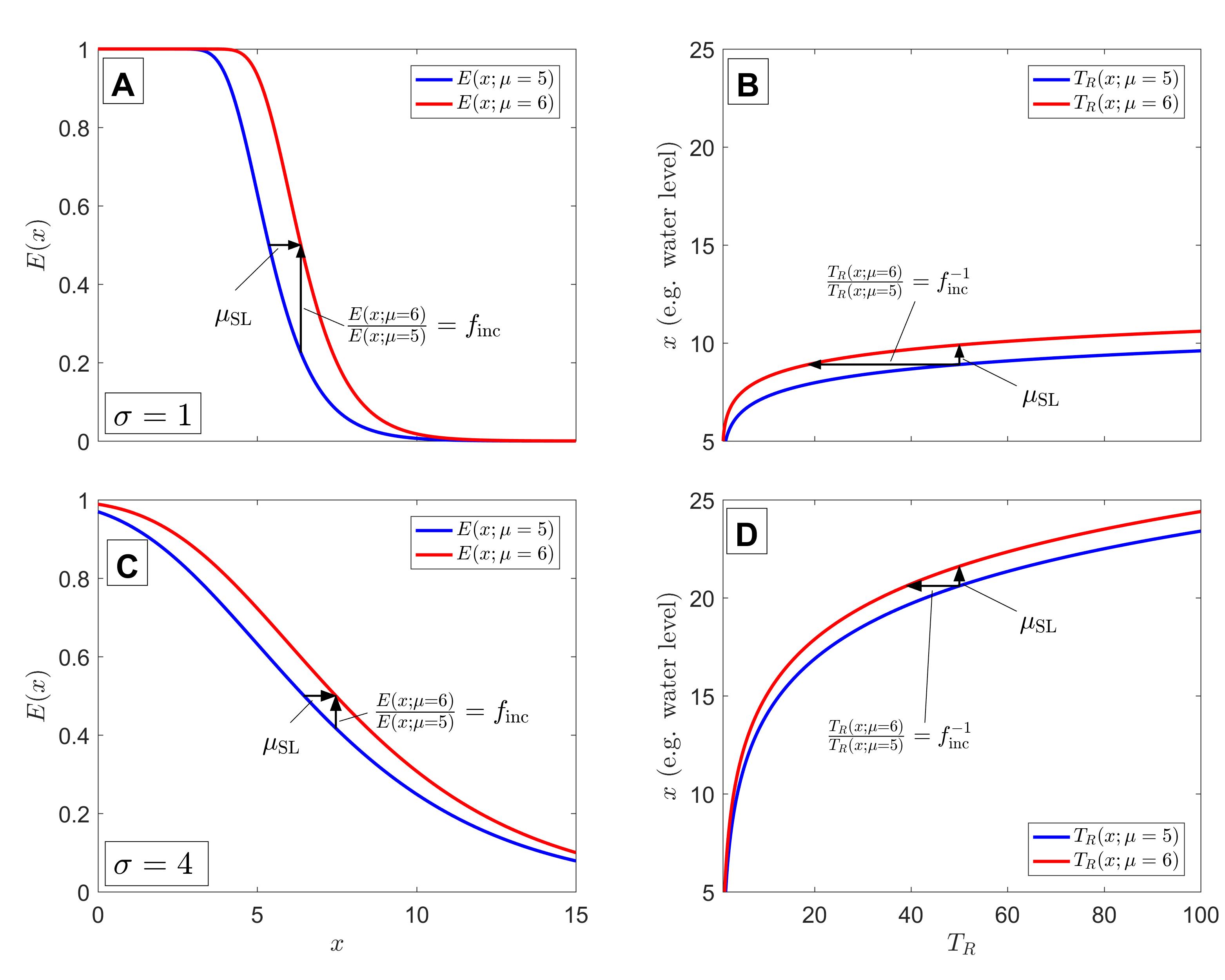


**Extended Data Figure 1 – Effects of scale parameter, $\sigma$, on changes in exceedance and return period with 1m SLR:** Panels A & B use $\sigma=1$ and panels C & D use $\sigma=4$. Blue lines are for distribution mean $\mu=5\text{ m}$ and red lines are for distribution mean$\mu+\mu_{SL}$, where $\mu_{SL}=1\text{ m}$. Comparing the upper panel with the lower, we see that a smaller value of the scale parameter, $\sigma$, results in a larger factor of increase in exceedance probability, $f_{\text{inc}}$. Thus SLR leads to an increase in frequency of extreme events that is governed by the *width* or scale parameter, $\sigma$, of the distribution. In regions with large values of $\sigma$, i.e. regions with significant variability in extreme water-level events, the relative frequency increase due to SLR will be less pronounced than in regions of small $\sigma$. Thus regions of large sea-level variability are buffered against future effects of SLR. On the other hand, regions where SLR is significant compared to the water-level variability are likely to experience significant frequency increases relative to present levels.


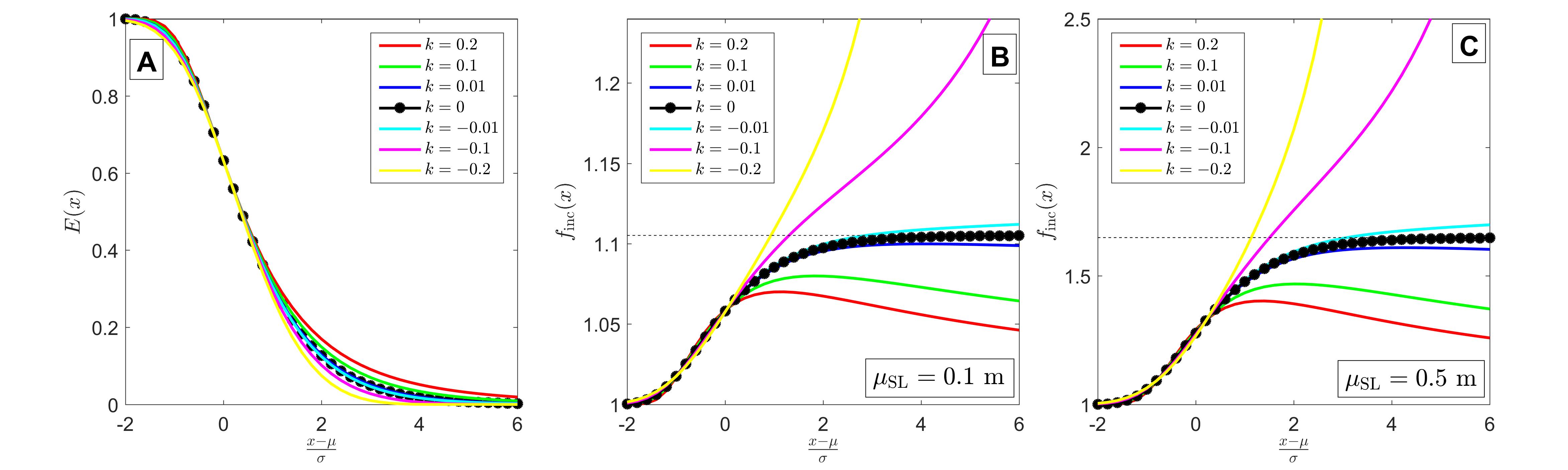


**Extended Data Figure 2 – Effects of shape parameter on exceedance probability and factor of increase, $f_{\text{inc}}$ :**

(A) Small values of $k$ cause the exceedance probability distribution to decay more rapidly than large values of $k$**,** but exceedance probability curves are similar for $-0.2\leq k\leq0.2$. (B) and (C) the factor of increase is sensitive to the changes in the shape parameter for . The functional shape of $f_{\text{inc}}$ is similar for $\mu_{\text{SL}}=0.1$ m and $\mu_{\text{SL}}=0.5$ m, although the magnitude (on the y-axis) is increased for the larger SLR case (C). The effect of shape parameter is thus similar to that of scale parameter: increasing the value of $k$ reduces the factor $f_{\text{inc}}$ and the effects of SLR. Thus regions with large (positive) values of $k$, i.e. regions whose extreme event probability decays slowly, will experience mild increases in flooding frequency due to SLR compared to regions of small (or negative) $k$.


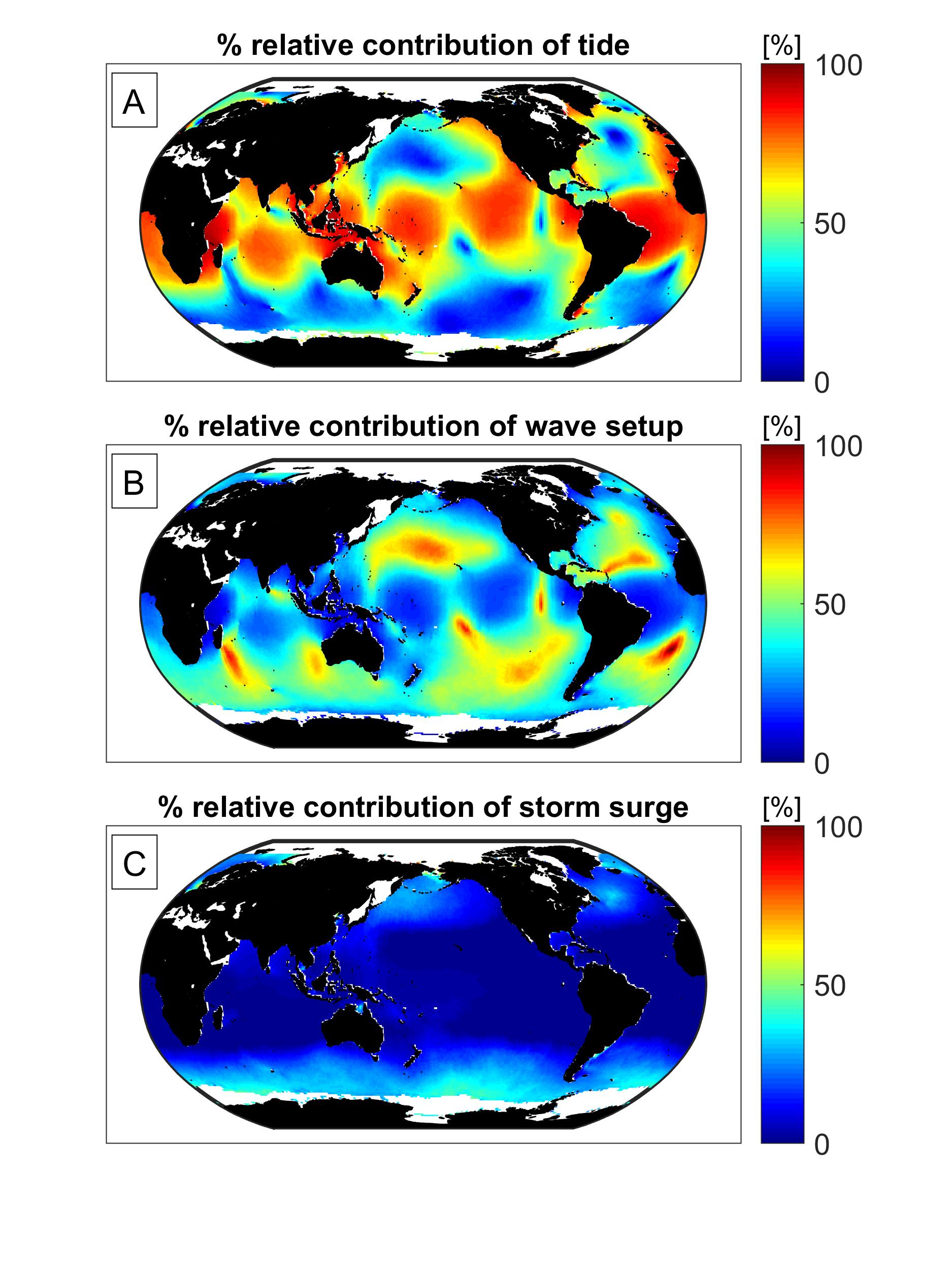


**Extended Data Figure 3 – Relative contributions of individual water-level components to the total water level (TWL). (A)** Tide **(B)** Wave setup **(C)** Storm surge. Calculations are based on the average relative contributions of the $n=63$ **(**top 3 annual maxima) of the 21-year time series of extreme wave setup, tide, and storm surge events to the total water level. For the majority of the globe, tide represents the largest contribution to the total water level. In the regions exposed to extratropical storms and regions near tidal amphidromes, wave setup also provides significant contributions to the total water level. Storm surge contributes little to the extremes of total water level (other than at high latitudes) at least in an average sense. The maps in this figure were made using Matlab 2016a (https://www.mathworks.com/products/matlab/).


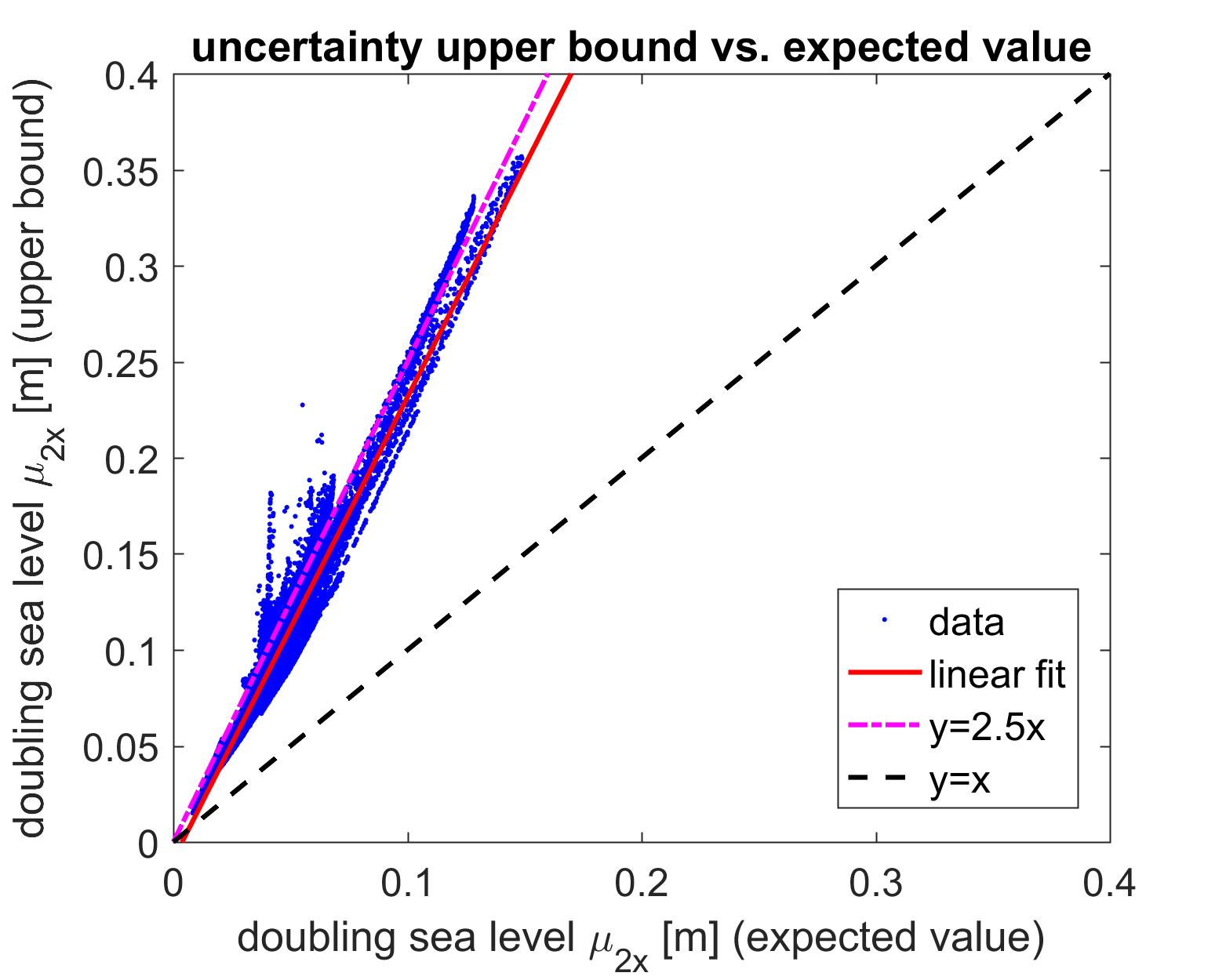


**Extended Data Figure 4 – The relationship between the expected value for the doubling sea level $\mu_{2\text{x}}$ and the upper bound of the 95% confidence interval for $\mu_{2\text{x}}$at each $1^{\circ}\times1^{\circ}$ grid point.** A Monte Carlo simulation with 100,000 random realizations based on the 95% confidence intervals of the GEV parameters is applied at each $1^{\circ}\times1^{\circ}$ grid point. The random sample of GEV parameters is used with Eq. (4) to calculate the empirical distribution for $\mu_{2\text{x}}$. The upper bound of the 95% confidence interval for(pink dashed line) is roughly 2.5 times its expected value (black dashed line).


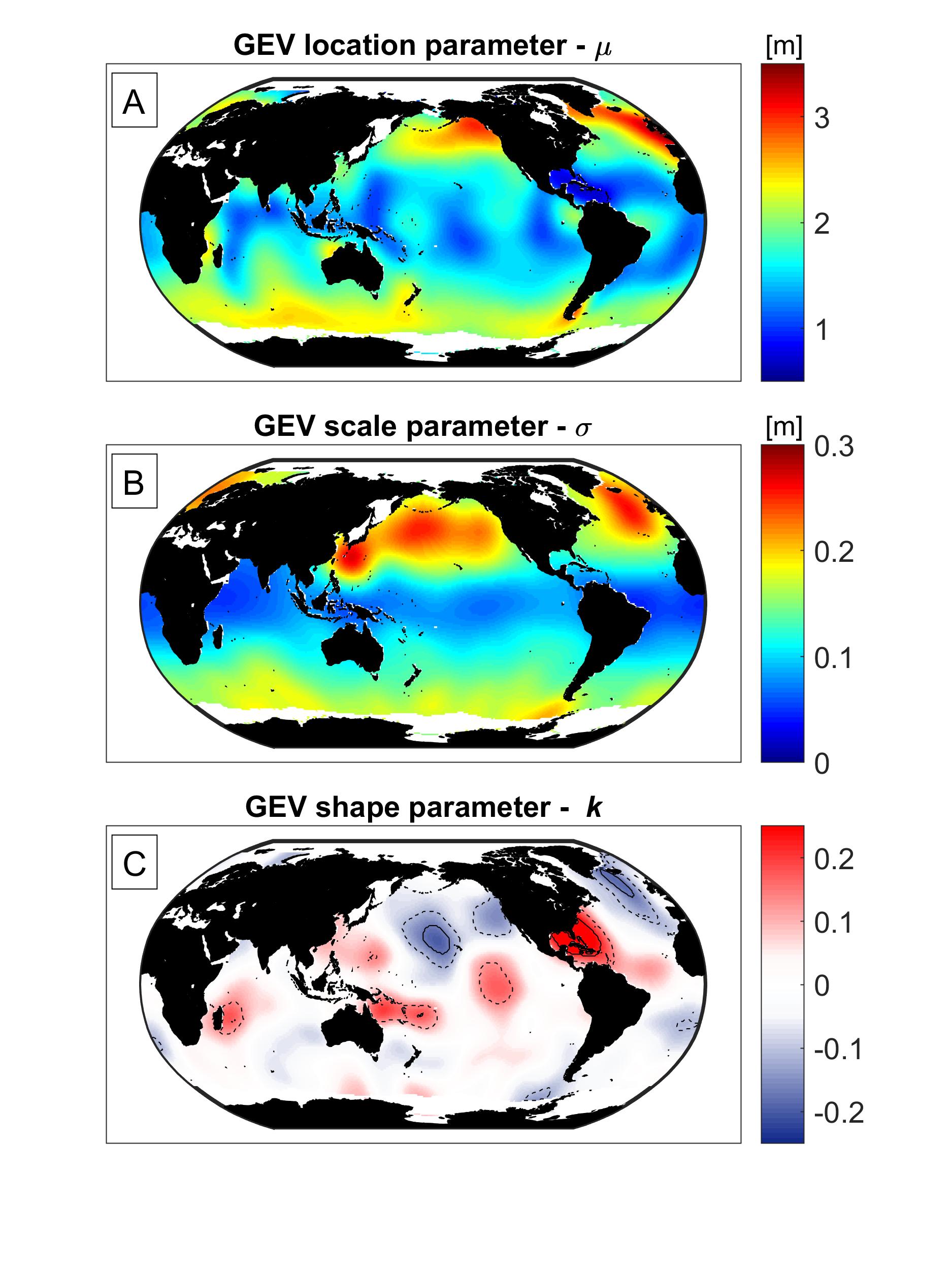


**Extended Data Figure 5 –** Global estimates of the location ($\mu$), scale ($\sigma$ ), and shape ($k$) parameters of the GEV distribution of extreme water-level (the sum of wave runup, tide, and storm surge) shown in panels A, B, and C, respectively. The dashed and solid lines in panel C represent contours of $k$ that are significantly different from zero at the 75% and 95% confidence levels, respectively. This figure illustrates the same analysis as shown in Figure 3 in the main text, but also includes the contribution of wave swash to TWL. The maps in this figure were made using Matlab 2016a (https://www.mathworks.com/products/matlab/).


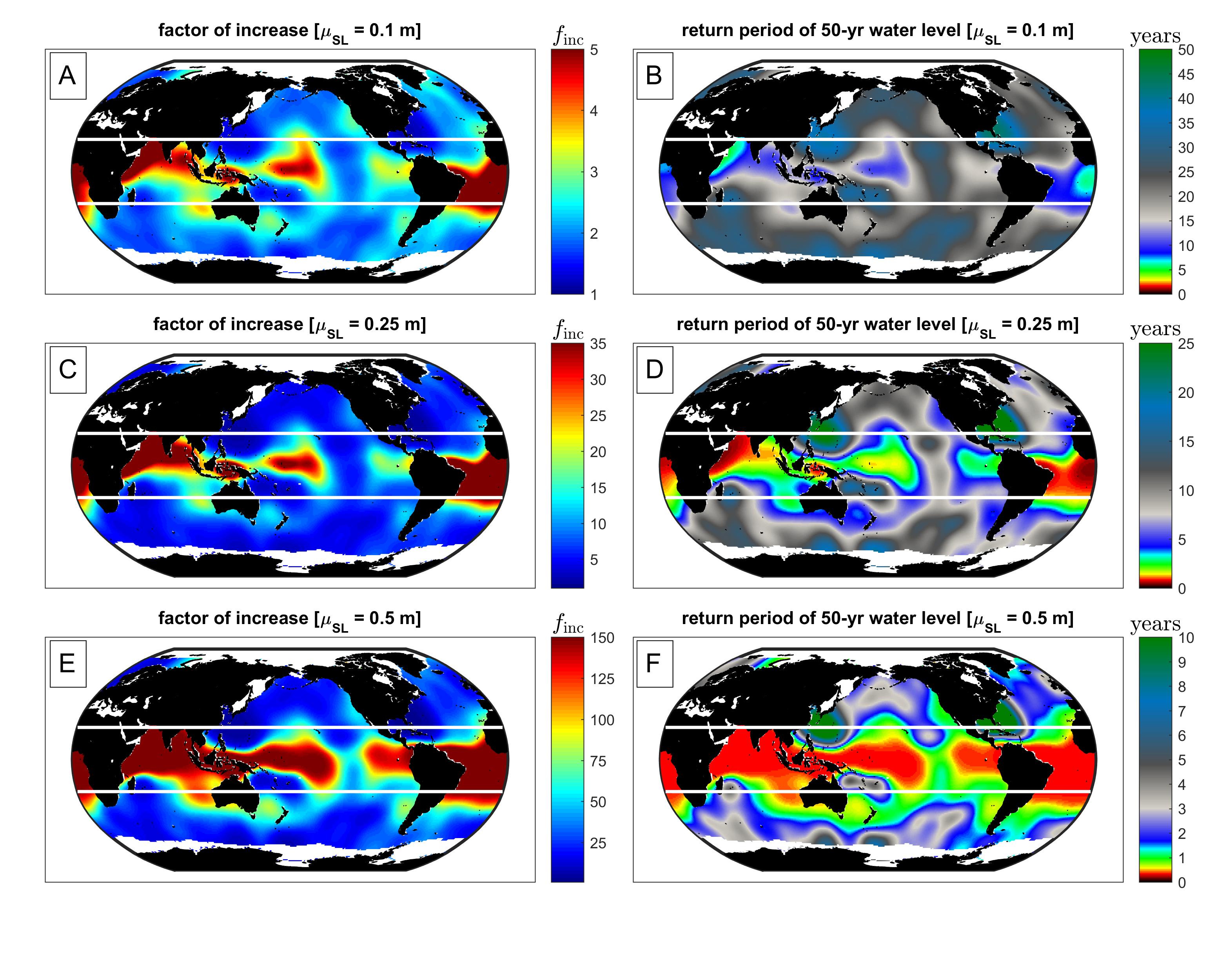


**Extended Data Figure 6 –** Global estimates of the expected factor of increase in exceedance probability, $f_{\text{inc}}$, and the future return period, $T_{R}$, of the 50-yr water level, for SLR projections: $\mu_{SL}=+0.1,\text{ +}0.25,\text{ +}0.5$ m. We note that the estimated increase in flooding potential is purely due to SLR and not due to changes in climate or storminess. White lines indicate the Tropic of Cancer and Tropic of Capricorn. This figure illustrates the same analysis as shown in Figure 4 in the main text, but also includes the contribution of wave swash to TWL. The maps in this figure were made using Matlab 2016a (https://www.mathworks.com/products/matlab/).


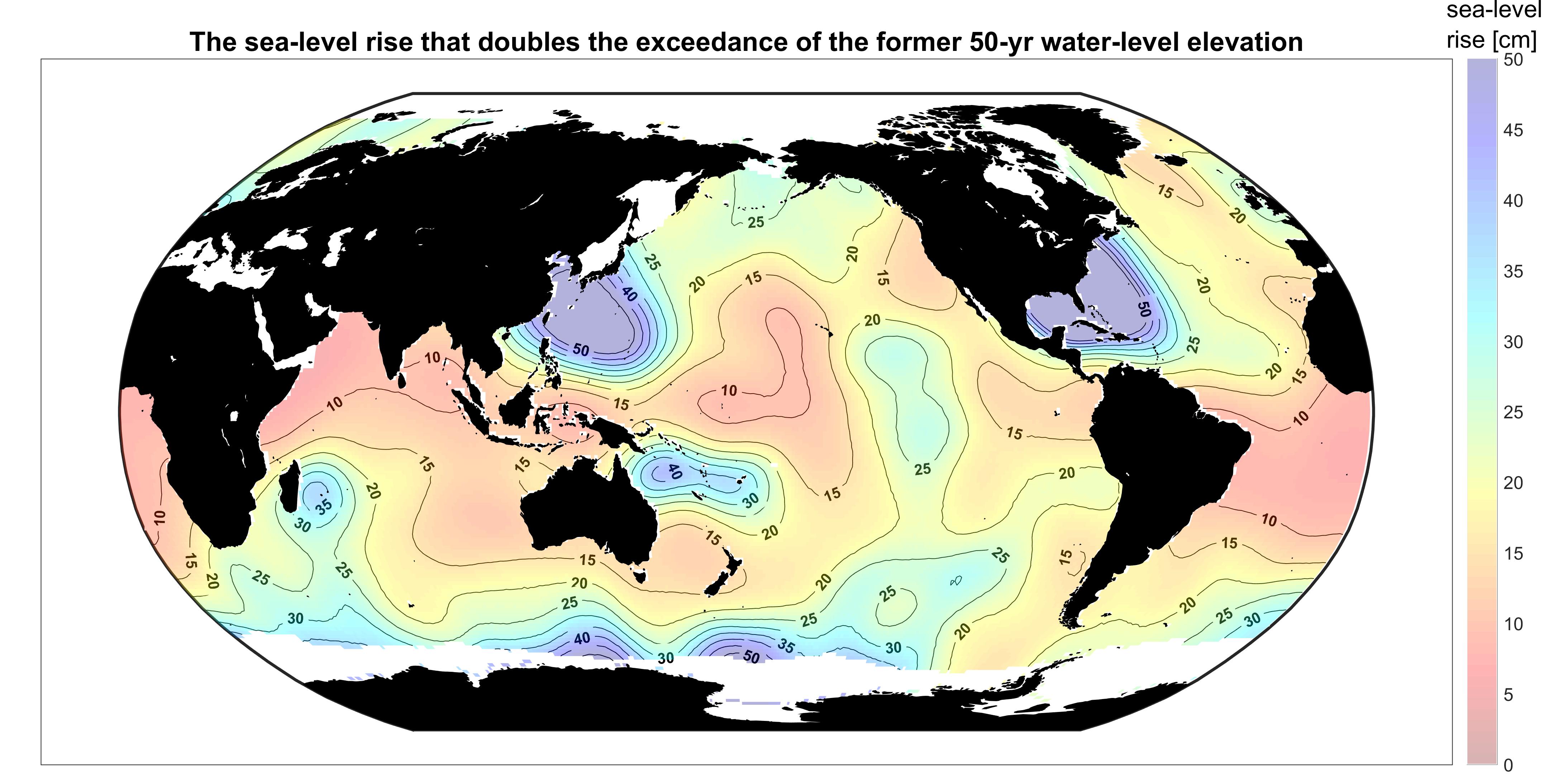


**Extended Data Figure 7 –** The upper bound of SLR that doubles the exceedance probability of the former 50-year water level. This SLR is the upper limit of a 95% confidence interval based on a Monte Carlo simulation of the GEV parameter estimates and their associated confidence bands (see Methods). Red areas represent regions particularly vulnerable to small amounts of SLR. This figure illustrates the same analysis as shown in Figure 5 in the main text, but also includes the contribution of wave swash to TWL. The maps in this figure were made using Matlab 2016a (https://www.mathworks.com/products/matlab/).


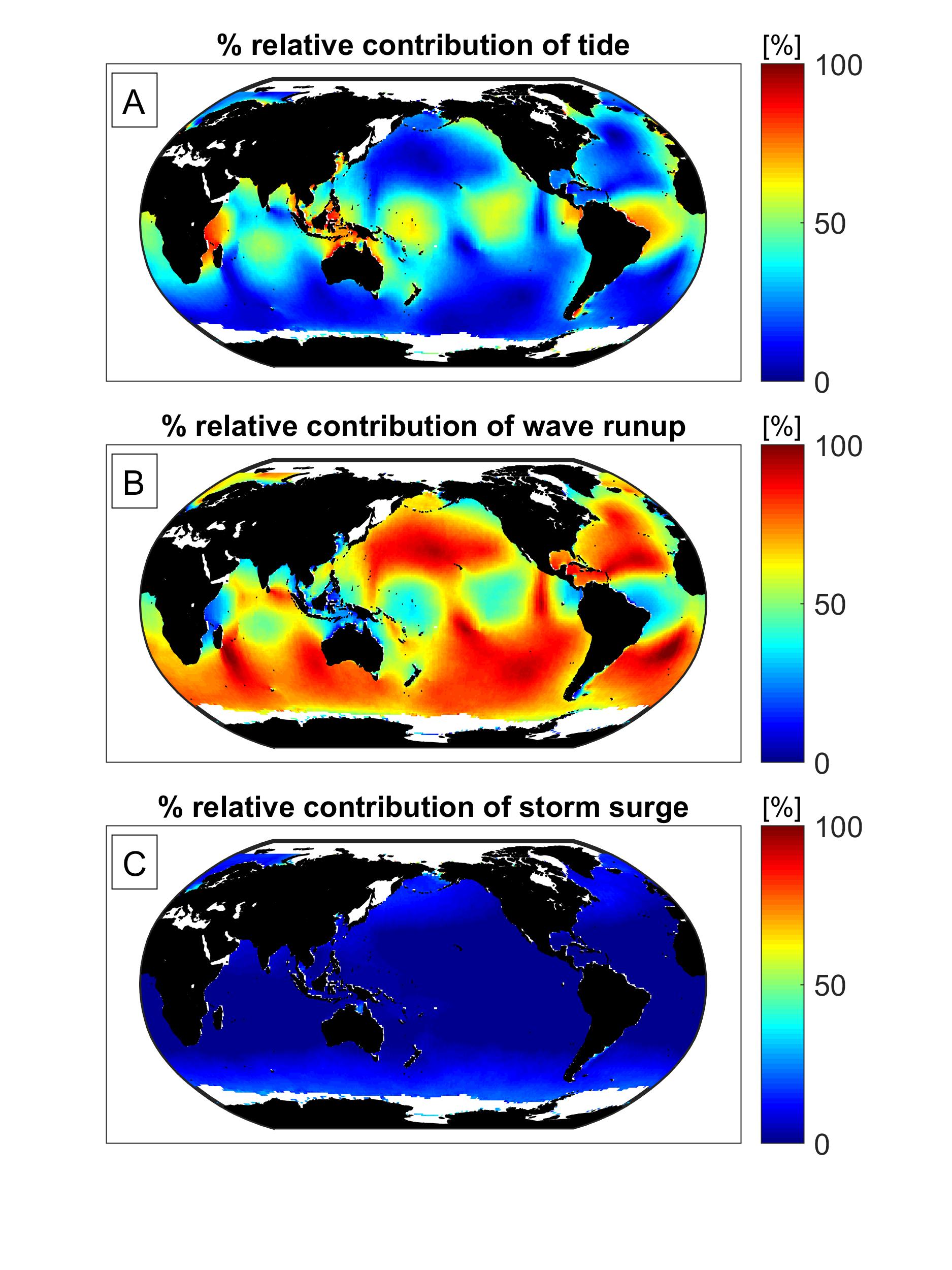


**Extended Data Figure 8 – Relative contributions of individual water-level components to the total water level (TWL). (A)** Tide **(B)** Runup (wave setup + wave swash) **(C)** Storm surge. Calculations are based on the average relative contributions of the $n=63$ **(**top 3 annual maxima) of the 21-year time series of extreme wave runup, tide, and storm surge events to the total water level. For the majority of the globe, wave runup represents the largest contribution to the total water level. This result is perhaps expected, since wave runup is a positive quantity whereas tide and storm surge are either positive or negative. In the Tropics and regions sheltered from wave activity, the tide also provides significant contributions to the total water level. Storm surge contributes little to the extremes of total water level (other than at high latitudes) at least in an average sense. This figure illustrates the same analysis as shown in Extended Data Figure 3, but also includes the contribution of wave swash to TWL. The maps in this figure were made using Matlab 2016a (https://www.mathworks.com/products/matlab/).
